# Intestinal Microbiota in Diabetes—Does the Pathomechanism and Diversity Depend on the Type of Diabetes and Coexisting Metabolic Disorders?

**DOI:** 10.3390/jcm15072604

**Published:** 2026-03-29

**Authors:** Clara Leśniak, Zuzanna Poczta, Maja A. Różycka, Olga M. Włoch, Agnieszka Podeszwa, Aleksandra Siejek, Agnieszka Dobrowolska, Agnieszka Zawada

**Affiliations:** 1 Student Scientific Society, Department of Gastroenterology, Dietetics and Internal Medicine, Poznan University of Medical Sciences, Rokietnicka Street 5E, 60-806 Poznań, Poland; 2 Department of Gastroenterology, Dietetics and Internal Medicine, Poznan University of Medical Sciences, Przybyszewskiego Street 49, 60-355 Poznań, Poland

**Keywords:** diabetes mellitus, gut microbiota dysbiosis, pathomechanism, glucose metabolism, lipids metabolism

## Abstract

Emerging evidence strongly suggests that gut microbiota dysbiosis plays a significant role in the development and progression of both type 1 and type 2 diabetes mellitus. Quantitative and qualitative changes in the intestinal microbiota’s composition are linked to these distinct pathomechanisms. In type 1 diabetes mellitus, dysbiosis is thought to initiate or accelerate the autoimmune destruction of pancreatic beta cells. This may occur through increased intestinal permeability, which allows microbial components and endotoxins to enter the systemic circulation. This exposure triggers inflammatory and autoimmune responses in individuals who are genetically predisposed. Conversely, in type 2 diabetes mellitus, gut dysbiosis contributes significantly to the characteristic metabolic derangements. Specific microbial shifts can lead to impaired energy metabolism, contributing to insulin resistance in peripheral tissues. Furthermore, dysbiosis is associated with the altered production of microbial metabolites, such as short-chain fatty acids, and the induction of low-grade chronic inflammation, which contribute to the pathogenesis of type 2 diabetes mellitus. Hyperglycemia, dyslipidemia and other metabolic changes also influence the gut microbiota. Understanding these type-specific microbial roles offers potential for novel diagnostic and therapeutic strategies.

## 1. Introduction

Diabetes is a group of metabolic disorders that result from an abnormal action or production of insulin [1]. The disease affects an increasing number of people, and its complications are a bigger public health problem every year [2]. In most cases, diabetes can be classified as type 1 (DM1) or type 2 (DM2). Still, we also distinguish other, less common types, such as maturity-onset diabetes of the young (MODY), gestational diabetes (GDM) [3], and diabetes secondary to pancreatic disease (DM3). DM1 is associated with the autoimmune destruction of pancreatic beta cells, leading to the cessation of insulin production. The pathomechanism of DM2 is a multifaceted process that results from hereditary and environmental interactions. Factors contributing to the disease include obesity or a sedentary lifestyle. In each case, chronic hyperglycemia results in the dysfunction of multiple organs, including the eyes, heart, kidneys, and nerves [4]. Gut microbiota dysbiosis is increasingly being considered as a factor in the pathomechanisms of both DM1 and DM2 [5]. The gut microbiota comprises approximately 1000 species of bacteria and plays a crucial role in maintaining human health. Although relatively stable in healthy adults, its composition undergoes both quantitative and qualitative changes in many diseases. The microbiota is involved in numerous physiological processes, including nutrient absorption, vitamin production, defense against pathogens, and the maturation of the immune system [6]. In diabetes, intestinal dysbiosis has been shown to significantly influence the regulation of glucose and lipid metabolism, as well as inflammatory and immune responses [7]. A deeper understanding of the role of intestinal dysbiosis in the pathomechanisms of diabetes may be key to more effective treatment of diabetic patients.

## 2. Aims of the Study

This narrative review aims to synthesize and critically assess the current evidence on the contribution of gut microbiota to the pathomechanisms of diabetes, and to examine whether microbial diversity and composition vary depending on the type of diabetes.

## 3. Materials and Methods

### 3.1. Search Strategy and Data Sources

This study was conducted as a narrative review based on the articles in English found on PubMed and Google Scholar. Original research and review articles published within the last 10 years were given priority. The search terms included various combinations of: “type 1 diabetes mellitus”, “type 2 diabetes mellitus”, “gut microbiota dysbiosis”, “intestinal dysbiosis”, “pathomechanism”, “glucose metabolism”, “lipids metabolism”, “diabetic dyslipidemia”, “inflammation”, “leaky gut syndrome”.

### 3.2. Study Selection and Inclusion Criteria

This study provides a comprehensive review of the literature on the role of gut microbiota in the pathogenesis of diabetes mellitus. Articles were selected based on their methodological quality and relevance. Both human and animal studies were considered, with particular emphasis on patient cohorts with diabetes. No limits regarding sample size were applied. The studies were included in this review if they focused on the relationship between the composition of the gut microbiota and diabetes, glucose metabolism, lipid metabolism, or leaky gut syndrome. If the studies were not relevant to the topic, not available in English, or lacked sufficient methodological detail, they were excluded from this review. In addition to the database searches, reference lists of the relevant articles were manually screened to identify additional studies that met the inclusion criteria. In total, 95 articles were included in the final analysis and six authors were involved in the screening process.

### 3.3. Data Collection and Synthesis

Data was collected from electronic sources. A qualitative synthesis approach was employed, in which key information from the included studies—such as study population, objectives, main results, and conclusions regarding changes in the gut microbiota—was systematically extracted. The extracted data were then thematically analyzed and interpreted.

### 3.4. Justification

This methodological approach was selected to provide a comprehensive and critical overview of the current state of research. It enables the synthesis of a broad range of findings, varying from human clinical cases to animal models and therefore offering an integrated perspective on clinical observations and underlying pathophysiological mechanisms. Furthermore, this approach not only summarizes existing knowledge but also helps identify critical gaps and helps inform future research directions.

## 4. Differentiation of the Microbiota According to the Type of Diabetes

The gut microbiota of individuals with diabetes differs from that of healthy individuals. Moreover, these differences vary depending on the type of diabetes.

### 4.1. DM1 and DM2

Research studies on the composition of the intestinal microbiome in patients with both DM1 and DM2 suggest several recurring microbial patterns. One of the most consistently reported findings is a reduction in butyrate-producing bacteria, suggesting an essential role for butyrate in blood glucose regulation.

Experimental studies in streptozotocin-induced DM1 mice have shown that butyrate exerts protective effects on pancreatic islet structure and function and can activate the expression of the Insulin1 and Insulin2 genes. The study demonstrated a decreased abundance of *Faecalibacterium prausnitzii* and *Roseburia intestinalis* in DM1 patients, which are considered key producers of butyrate in the gut microbiota [5]. A similar pattern has been observed in DM2, where reductions in *Faecalibacterium prausnitzii*, *Roseburia intestinalis*, and *Roseburia inulinivorans* have been documented [8]. These findings suggest that depletion of butyrate-producing microorganisms may represent a shared microbial signature across both types of diabetes.

Another bacterial group frequently reported to be reduced in both DM1 and DM2 is the genus *Akkermansia* [9,10]. Of particular interest is *Akkermansia muciniphila*, whose abundance has been shown in some studies to decline even before the onset of DM2, suggesting that changes in the gut microbiota may precede clinical disease development [11,12]. This bacterium is considered beneficial due to its role in the maintenance of intestinal mucus integrity, reduction in intestinal permeability, and its anti-inflammatory and immunomodulatory effects. Moreover, its presence has been associated with improved insulin sensitivity, further highlighting its potential importance in metabolic health and the pathogenesis of diabetes [13].

In both DM1 and DM2, a decline in bacteria of the genus *Bifidobacterium* can be observed [14,15]. In DM1, a decrease in the two dominant species of the genus *Bifidobacterium*, namely *B. adolescentis* and *B. pseudocatenulatum*, was observed [15,16]. In DM2, some *Bifidobacterium* species, such as *B. adolescentis*, are in turn being studied for use in probiotics for patients. Research on animals has shown that a higher number of *B. adolescentis* strains can reduce insulin resistance [17]. A substantial number of studies consistently report a reduced abundance of these bacteria in individuals with diabetes. *Bifidobacterium* species are well documented to exert beneficial effects on the host, including modulation of the immune response, such as lowering the levels of pro-inflammatory cytokines and increasing the number of CD4+ T lymphocytes and IgA-producing plasma cells. In addition, these bacteria contribute to host health through their metabolic activity, particularly by fermenting otherwise indigestible dietary carbohydrates and thereby generating metabolites that can support host nutrition and gut homeostasis [13].

Data from the TEDDY Study, which followed children from 3 months of age, indicate that early alterations in *Bifidobacterium* are species-specific: healthy children had higher levels of *Bifidobacterium dentium*, whereas those who later developed type 1 diabetes showed increased *Bifidobacterium pseudocatenulatum*. These changes suggest that diabetes-associated microbiota alterations may involve both increases and decreases in particular species [18]. Although certain species such as *B. pseudocatenulatum* may be elevated in early or preclinical stages, their abundance typically declines as the disease progresses.

With regard to Bacteroides bacteria, studies in patients with type 2 diabetes (T2D) have yielded conflicting results. Some recent studies indicate an increased abundance of these bacteria in T2D [13,19], while other studies report a decrease in their abundance in patients [20,21].

A similar contradiction can be observed in the case of bacteria of the Firmicutes. Some studies indicate a reduced abundance of these bacteria in patients with type 2 diabetes [13,22]. Consequently, the Bacteroidetes-to-Firmicutes ratio changes, which may serve as a marker of the disease [23]. However, some studies have reported an increased abundance of these bacteria [21]. The inconsistencies observed across studies may stem from differences in methodologies, as well as variations in the populations examined. Factors such as diet, lifestyle, and age of the participants can also significantly influence the gut microbiota composition, potentially contributing to the divergent findings.

In contrast, for DM1, most studies indicate an increase in the abundance of Bacteroidetes. Studies have shown that children with increased numbers of this type of bacteria show progressive autoimmunity of pancreatic β-cells [24]. Some studies show that there is an increase mainly in the genus *Bacteroides*, especially the species *Bacteroides ovatus* [25], as well as *Bacteroides stercoris*, *Bacteroides fragilis* and *Bacteroides intestinalis* [26]. This type of diabetes has also been found to have higher concentrations of bacteria of the genus *Prevotella*, which, like Bacteroidetes, can activate inflammatory pathways and reduce the integrity of the intestinal mucosa [27].

Studies investigating the abundance of *Lactobacillus* in DM2 have reported inconsistent findings. In patients with type 2 diabetes, both increased and decreased levels of *Lactobacillus* have been described in different studies [13,23]. However, recent research indicates that interventions involving prebiotics that promote *Lactobacillus* growth may contribute to restoring gut homeostasis, reducing inflammation, and improving metabolic health in individuals with type 2 diabetes [12,28]. These findings suggest that *Lactobacillus* may play a protective role in the context of type 2 diabetes.

Alterations have also been described in members of the genus *Clostridium*. Studies conducted in European and Chinese cohorts demonstrated reduced abundance of several *Clostridium* species in patients with DM2. The abundance of these bacteria correlated negatively with plasma glucose, HbA1c, insulin, and C-peptide levels, and positively with adiponectin and HDL concentrations [29].

**Figure 1:**
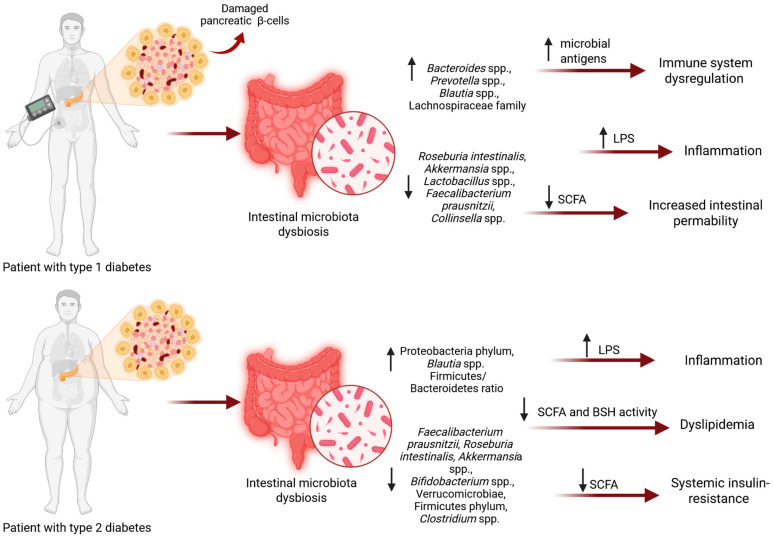
The role of gut microbiota dysbiosis in the pathogenesis of DM1 and DM2. Abbreviations: LPS, bacterial lipopolysaccharide; SCFA, short-chain fatty acid; BSH, bile salt hydrolase; ↑, increased abundance; ↓, decreased abundance.

Differences have also been observed in the abundance of bacteria belonging to the genus *Blautia*, which appears to be increased in individuals with both DM1 and DM2. In addition, patients with DM1 have been reported to show reduced levels of bacteria from the genus *Collinsella* and increased levels of members of the Lachnospiraceae family. Levels of *Collinsella* have been positively correlated with plasma insulin concentrations, whereas increased abundance of Lachnospiraceae has been associated with impaired glucose metabolism [30] (Figure 1). Gut microbiota differences and their functional impacts in diabetes are presented in Table 1. 

### 4.2. MODY

Alterations in the gut microbiota are also observed in MODY-HNF1A. Evidence indicates that these changes differ from those reported in DM2 and are characterized by a higher abundance of bacteria belonging to the order Turicibacterales. Furthermore, comparative analyses with healthy control subjects have demonstrated that the phylum Proteobacteria is more abundant in individuals with DM2, whereas the phylum Bacteroidetes is less abundant in both DM2 and MODY-HNF1A [31]. A different study comparing children with DM1 and MODY2 diabetes showed that MODY2 was associated with increased *Prevotella* and reduced *Ruminococcus* and *Bacteroides*, whereas DM1 was characterized by higher abundances of *Bacteroides*, *Ruminococcus*, *Veillonella*, *Blautia*, and *Streptococcus*, alongside lower levels of *Bifidobacterium*, *Roseburia*, *Faecalibacterium*, and *Lachnospira* [24].

### 4.3. GDM

GDM is associated with distinct alterations in gut microbiota composition, as demonstrated by a meta-analysis synthesizing data from seven studies. Specifically, GDM is characterized by a reduced abundance of *Bifidobacterium*, *Lactobacillus*, *Bacteroides*, and Bacteroidetes, alongside an increased abundance of *Enterobacter*, *Enterococcus*, and *Fusobacterium* [32]. Moreover, GDM-related gut dysbiosis is reflected by a lower phylogenetic diversity (PD) index, accompanied by an increased abundance of Lachnospiraceae and a decreased abundance of Enterobacteriaceae and Ruminococcaceae [33]. Other bacteria reported to be enriched in GDM include *Parabacteroides distasonis* and *Klebsiella variicola* [34].

Despite numerous studies on the microbiota composition of individuals with diabetes, contradictory results persist. It is essential to conduct further research on this issue for its potential application in the treatment of this disease. Modification of the gut microbiota could be a relatively simple intervention capable of improving metabolic control in diabetes. However, this requires the identification of specific microbial strains that influence glycaemic regulation.

## 5. Effects of the Microbiota on Glucose Metabolism

The intestinal microbiota plays a critical role in regulating various metabolic and hormonal functions through the fermentation of dietary fiber and starch. This fermentation produces short-chain fatty acids (SCFAs), including acetate, propionate, and butyrate, which influence multiple metabolic pathways and are particularly important for glucose and lipid metabolism [35].

In healthy individuals, SCFAs regulate glucose metabolism at several levels across different tissues and organs. In the liver, they promote glycogen synthesis, enhance AMPK activity and insulin sensitivity, and suppress gluconeogenesis and glycolysis. In the pancreas, SCFAs stimulate insulin secretion while inhibiting glucagon release. In skeletal muscle, they increase GLUT4 transporter expression and glycogen synthesis while reducing glycolysis. Within the gut, SCFAs contribute to improved insulin sensitivity. Additionally, SCFAs promote satiety and reduce appetite, which helps prevent excessive weight gain. Together, these effects support proper glucose homeostasis and help reduce the risk of obesity and diabetes [35].

A high diversity of the gut microbiota has an impact on maintaining adequate host health. A reduced or less diverse microbiota will be unable or less able to ferment fiber, and the number of SCFAs formed in this process may adversely decrease (e.g., butyrate) or increase (e.g., propionate), compromising the regulation of the metabolic pathways they control [36].

The results of numerous studies, both clinical and based on animal models, strongly suggest that the composition of the intestinal microbiota may influence metabolic disorders such as insulin resistance and diabetes. According to this hypothesis, patients with prediabetes or DM2 often exhibit intestinal dysbiosis compared to healthy individuals. For example, in two independent studies conducted by Zhang’s [37] and Wu’s [38] teams on three study subgroups (healthy subjects with standard glucose tolerance, subjects with prediabetes, and subjects with DM2), it was observed that subjects with poor glucose tolerance (subgroups 2 and 3, respectively) had a lower amount of butyrate-producing bacteria in the composition of their gut microbiota, which is essential for regulating glucose metabolism.

Similar conclusions were reported by Zhu and Goodarzi [39], who showed that gut microbiota composition can affect blood glucose levels. They found that lower bacterial diversity is associated with insulin resistance and higher obesity, and that DM2 and higher blood glucose levels are linked to reduced relative abundance of bacteria of Firmicutes and Clostridia, the genera involved in butyrate production for glucose regulation. Decreased levels of *Akkermansia muciniphila*, *Verrucomicrobiae*, and *Faecalibacterium prausnitzii* were associated with impaired insulin sensitivity [37], while the abundance of Proteobacteria genus increased significantly with insulin resistance [40]. In their study, Zhang et al. [37] further demonstrated that reduced numbers of *Akkermansia muciniphila* are associated with a higher risk of carbohydrate metabolism disorders, suggesting that the amount of *A. muciniphilia* may serve as a specific marker of impaired glucose tolerance. Wang et al. [41] reported that individuals with insulin resistance show increased capacity to synthesize branched-chain amino acids (BCAAs) and decreased ability to transport BCAAs into bacterial cells, with lower BCAA production associated with the presence of *Prevotella copri* and *Bacteroides vulgatus*.

Additionally, a study by Wen and colleagues [42] demonstrated that interactions between gut bacteria and the host immune system can either reduce or increase type 1 prediabetes, highlighting the importance of proper microbiota composition for human health. Peng et al. [43] reached similar conclusions, showing that both qualitative and quantitative disorders of gut bacteria affect immune system proteins.

Based on numerous studies on the intestinal microbiota composition and the imbalance between different bacterial species, certain bacteria appear to contribute to weight gain by inducing the expression of genes involved in glucose and lipid metabolism. In addition to studies on the effects of diet, prebiotics, and probiotics on the composition of the microbiota, fecal microbiota transplantation (FMT) method has also been investigated. The fecal microbiome from healthy donors is transplanted into individuals with metabolic disorders [44]. In a study by Kootte et al. [45], the transplantation of FMT from lean donors significantly reduced HbA1c levels and improved insulin sensitivity in the recipients; however, these effects were not permanent, and HbA1c levels returned to baseline after 18 months. Conversely, another study on the impact of FMT on the composition of the intestinal microbiota in transplant subjects observed increased levels of butyrate-producing bacteria (e.g., *Ruminococcus bromii* and *Roseburia intestinalis*), which may enhance insulin sensitivity by regulating GLP-1 and influencing gluconeogenesis in the gut [46].

These examples suggest that gut microbiota composition, along with the SCFAs and BCAAs it produces, has a significant impact on insulin resistance and the regulation of glucose metabolism.

## 6. Effect of Microbiota on Lipid Metabolism

One of the conditions commonly associated with diabetes is dyslipidemia, which in the context of diabetes can be viewed from three perspectives. First, lipoprotein abnormalities include elevated triglycerides (hypertriglyceridemia) and reduced HDL-C levels. Second, qualitative abnormalities involve increased levels of large VLDL from the very low-density subfraction 1, elevated levels of small, dense LDL, increased triglycerides in LDL and HDL, glycation of apolipoproteins, and greater susceptibility of LDL to oxidation. Third, kinetic abnormalities include increased VLDL1 production, decreased VLDL catabolism, and increased HDL catabolism [43]. One of the conditions commonly associated with diabetes is dyslipidemia, which in the context of diabetes can be viewed from three perspectives. First, lipoprotein abnormalities include elevated triglycerides (hypertriglyceridemia) and reduced HDL-C levels. Second, qualitative abnormalities involve increased levels of large VLDL from the very low-density subfraction 1, elevated levels of small, dense LDL, increased triglycerides in LDL and HDL, glycation of apolipoproteins, and greater susceptibility of LDL to oxidation. Third, kinetic abnormalities include increased VLDL1 production, decreased VLDL catabolism, and increased HDL catabolism [47]. Patients with DM2 frequently exhibit gut microbiota dysbiosis. Reduced bacterial diversity has been linked to insulin resistance, inflammation, obesity, and impaired lipid profiles. Cotillard et al. reported that in obese individuals, reduced microbiota diversity was associated with elevated total cholesterol and triglycerides [48]. Similarly, Le Chatelier et al. found that individuals with low microbiota gene counts had higher triglyceride levels and lower HDL levels [49]. Lipid metabolism is further influenced by microbiota-derived metabolites, including SCFAs and bile acids, which affect dyslipidemia through cholesterol regulation [50].

SCFAs—primarily acetate (C2), propionate (C3), and butyrate (C4)—are produced by bacterial fermentation of indigestible carbohydrates. These SCFAs are absorbed into the bloodstream and act on peripheral tissues such as the liver, adipose tissue, and skeletal muscle. They bind to GPR43 (free fatty acid receptor 2, FFA2) and GPR41 (free fatty acid receptor 3, FFA3) on intestinal L cells, stimulating secretion of GLP-1 and PYY [51]. In the liver, acetate and butyrate serve as primary substrates for de novo lipogenesis and cholesterol synthesis, while propionate is an effective inhibitor of these processes. Under physiological conditions, acetate and propionate inhibit adipose tissue lipolysis [52], thereby reducing the transport of free fatty acids (FFAs) from adipose tissue to the liver. In DM2, reduced SCFA production due to diminished prevalence of SCFA-producing bacteria decreases this inhibitory effect, allowing more FFAs to reach the liver. This can lead to hepatic steatosis and altered lipid profiles, including overproduction of VLDL, which contributes to insulin resistance and abnormal lipid metabolism [53].

Bile acids also play a key role in lipid regulation. Approximately 95% of conjugated bile acids are reabsorbed from the small intestine via the portal circulation and secreted again by the liver. Intestinal bacteria with active bile salt hydrolase (BSH) then detach glycine or taurine from conjugated bile acids and form secondary (unconjugated) bile acids. Metagenomic analyses indicate that BSH activity is widespread among human gut bacteria, including *Lactobacillus*, *Bifidobacterium*, *Clostridium*, and *Bacteroides* [54]. BSH activity enhances bile acid elimination, promoting cholesterol catabolism [55]. Bacteria also modulate the activity of the farnesoid X receptor (FXR), which regulates cholesterol metabolism [50] and lipid absorption [56], as well as bile acid recycling, hepatic fatty acid, triglyceride synthesis, and VLDL production [57]. In patients with DM2, lower levels of secondary bile acids [58] and microbiome dysbiosis have been observed. Bile acids influence lipid metabolism by interacting with FXR as well as with Takeda G protein-coupled receptor 5 (TGR5), a membrane receptor [59]. FXR activation stimulates glycogen synthesis and enhances insulin sensitivity in obese mice [60], whereas FXR deficiency leads to glucose intolerance, reduced insulin sensitivity, elevated triacylglycerides, cholesterol, and bile acids, resulting in hepatic fat accumulation. These findings demonstrate a molecular link between bile acids and lipid metabolism [57]. Overall, TGR5 is expressed in brown adipose tissue and the small intestine, where bile acids act as agonists. Activation of TGR5 has been shown to influence lipid metabolism directly [61] and to induce GLP-1 secretion from intestinal L cells, improving liver and pancreatic function in obese mice, with potential downstream effects on lipid synthesis and storage.

Taken together, these findings show the profound impact of reduced gut microbiota diversity and their metabolites on lipid metabolism. Dysbiosis can influence lipid homeostasis indirectly by promoting insulin resistance or directly through metabolite-mediated regulation of lipid synthesis, transport, and storage.

## 7. Connection Between Leaky Gut Syndrome, Intestinal Microbiota, Inflammation, and Diabetes

The surface of the intestine is inherently exposed to various antigens, such as food antigens, food-borne pathogens, and commensal microorganisms. Intestinal epithelial cells have developed unique barrier functions that prevent the translocation of potentially harmful antigens into the body. Disruption of the epithelial barrier increases intestinal permeability, leading to leaky gut syndrome (LGS). Many lines of research suggest that epithelial barrier dysfunction may result from the loss of beneficial species due to gut dysbiosis. It is known that both exogenous factors (e.g., diet and medication) and endogenous factors (e.g., antimicrobial peptides, sIgA, and the mucin layer) influence the gut microbiota community.

Furthermore, mutations in several genes (i.e., NOD2 and XBP1) and environmental stressors (such as obesity and radiation) can cause dysfunction of Paneth cells, impairing the secretion of antimicrobial peptides and leading to dysbiosis. Adhesion of microbes to the epithelium can initiate a sequence of inflammatory responses by activating signal transduction through TLR and zonulin signaling, leading to loss of tight junction (TJ) integrity. Increasing evidence shows that microbial metabolites can serve as exogenous regulators of TJ barriers. For example, butyrate, an SCFA, strengthens the TJ barrier by inducing a hypoxic response. In mice that spontaneously develop DM2, epithelial dysfunction is accompanied by insufficient representation of the major producer of butyrate, *Faecalibacterium prausnitzii* [62].

SCFAs such as acetate or butyrate also affect the function of macrophages, T and B lymphocytes, and dendritic cells. Thus, they stimulate the immune system to respond appropriately to pathogens [63]. At the same time, they have been shown to inhibit the production of proinflammatory cytokines and induce the formation of Treg lymphocytes [64]. In DM2, a decrease in acetate- and butyrate-producing bacteria, such as *Bifidobacterium*, is documented. From this, it follows that the lack of SCFA-producing bacteria will also contribute to inflammation. Another proinflammatory factor may be bacterial lipopolysaccharide (LPS), an endotoxin that forms part of the cell wall of Gram-negative bacteria [65]. LPS can enter the circulation through the intestinal barrier, which has reduced integrity due to changes in the intestinal microbiome, causing metabolic endotoxemia [66]. LPS is a ligand for the toll-like receptor (TLR), which is found on various cell types, including macrophages. The binding of LPS to TLR4 induces the expression of several hundred genes. This, in turn, causes the secretion of cytokines such as IL-1, IL-6, and TNF-α, which induce an inflammatory response [67]. Inflammation, meanwhile, can affect insulin resistance. For example, it has been shown that TNF-α can decrease insulin receptor tyrosine kinase activity. In mouse adipocytes, TNF-α induced serine phosphorylation of insulin receptor substrate-1 (IRS-1) into an insulin receptor inhibitor with tyrosine kinase activity [68]. An experiment in mice, involving subcutaneous infusion of LPS for 4 weeks, showed that insulin resistance in the liver appears, as well as an increase in inflammatory markers. Notably, the increase in intestinal LPS concentrations also occurred when mice were fed a high-fat diet, which can be explained by the fact that LPS is transported with chylomicrons. Also worth mentioning is the effect of specific bacterial species on LPS concentrations and on inflammation. It was studied that higher levels of bacteria of the genus *Bifidobacterium* correlated negatively with endotoxemia and positively with improved glucose tolerance [69]. In contrast, the species *Faecalibacterium prausnitzii*, a decrease which occurs in DM2 subjects, negatively correlates with an increase in inflammatory markers [70]. Additionally, microbiota containing LPS and metabolic endotoxemia are associated with the initiation of obesity [71].

Less information is available on inflammation in DM1, but it has also been shown to be associated with a proinflammatory state [72]. Clinical reports suggest that LGS contributes to autoimmune diseases such as DM1, multiple sclerosis, rheumatoid arthritis, and celiac disease [62]. Studies have shown that in DM1, levels of TLR2 and TLR4 ligands, also known as endotoxins, are significantly elevated. This, as in DM2, contributes to increased activity of these receptors and induction of inflammation [73]. Another study reports significantly increased hsCRP in children with DM1, which also indicates the presence of low-grade inflammation [74]. Experimental observations have confirmed that LGS promotes genetically induced autoimmunization. Induction of LGS by administering Sodium Dodecyl Sulfate (SDS) (a laxative medication) leads to the activation of autoreactive T cells in the gut of a mouse model with DM1. Ultimately, this response triggers diabetes. However, antibiotic therapy has inhibited the disease’s progression. The accumulated evidence suggests that cross-reactivity with microbial antigens can induce autoimmune reactions. There is also compelling evidence of a connection between the oral and gut microbiota. In particular, dysbiosis in the oral cavity and the use of proton pump inhibitors facilitate the translocation of naturally occurring oral bacteria into the gut. Significantly, *Porphyromonas gingivalis*, a bacterium that causes periodontal disease, may predispose hosts to systemic inflammation and autoimmunization by inducing LGS [57].

Notably, studies have shown a link between the development of other inflammatory diseases, such as inflammatory bowel disease and diabetes. One of them, using the National Health Insurance database in South Korea, showed that patients with Crohn’s disease have a significantly increased risk of developing diabetes, regardless of the use of steroid drugs. The authors suggest considering regular blood glucose testing in these patients [75]. Similar results were obtained in a nationwide cohort study conducted in Denmark, which found that people with Crohn’s disease or ulcerative colitis have a significantly increased risk of developing DM2 [76]. In addition, similar to diabetes, Crohn’s disease is associated with intestinal dysbiosis, characterized by a decrease in species such as *Faecalibacterium prausnitzii* and *Bifidobacterium adolescentis*, among others [77]. This may indicate that dysbiosis is a pathogenic factor in both of these diseases. These studies may be relevant to treatment selection for patients with concomitant diseases and may suggest a common pathogenesis underlying these conditions. A summary of prior findings is presented in Figure 1.

Given the potential central role of the gut microbiota and LGS in the pathogenesis of diabetes, therapeutic interventions targeting the microbiota are becoming increasingly relevant. These interventions include: probiotic supplementation to rebuild SCFA-producing microbiota, prebiotics to promote the growth of beneficial gut bacteria, FMT, and reducing the use of proton pump inhibitors.

## 8. Gestational Diabetes Mellitus and Neonatal Microbiome

It is widely recognized that the health status of the mother significantly influences fetal development and overall neonatal health. Maternal disorders occurring during pregnancy may affect the fetus and lead to further health complications for both the mother and the child. One condition that deserves particular attention is the impact of GDM on the neonatal microbiome. Evidence suggests that during pregnancy, the vaginal, oral, placental, and gut microbiota undergo significant changes [78]. Consequently, the occurrence of dysbiosis in the maternal body may alter the maternal metabolic profile and contribute to gestational complications, thereby affecting fetal health.

The fetus is thought to be first exposed to microorganisms in utero; however, the most significant microbial colonization occurs during vaginal delivery through the birth canal [79,80]. After birth, breast milk further supports the establishment and development of the infant’s gut microbiota [81].

Significant alterations in the gut microbiome have been observed in the offspring of mothers with GDM. These include reduced α- and β-diversity, as well as changes in the relative abundance of specific bacteria [82]. Neonates born to mothers with GDM often exhibit gut microbiota characterized by increased abundance of pro-inflammatory bacteria and lower microbial diversity compared with those born to healthy mothers [83,84]. Such alterations may increase the risk of developing metabolic disorders, including diabetes.

A study of Crusell et al. [83] also concluded that the microbiota composition in the offspring of mothers with GDM resembles the gut microbiota patterns observed in childhood obesity and in adults with DM2. This observation may be related to the known predisposition to DM2 among children born to women diagnosed with GDM during pregnancy [85]. Although studies indicate that women with GDM have an increased risk of developing DM2 later in life, and that their offspring may also be at increased risk [85], further research is required to determine a direct link between inherited microbial dysbiosis in infants and the subsequent development of DM2.

Alterations in gut microbiota composition, particularly during the early days of life, have also been associated with immune dysregulation and an increased risk of chronic diseases, including autoimmune disorders [86]. Studies have shown that children with bacterial dysbiosis may progress to the clinical onset of DM1, often accompanied by intestinal inflammation, well before the development of hyperglycemia [87].

The proper development and maturation of a newborn’s gut microbiota are essential for the normal functioning of multiple physiological systems. To reduce the potential transmission of dysbiosis from mothers with GDM, several preventive strategies may be considered. One approach involves minimizing factors that disrupt the maternal microbiota. This may include improving gut microbiota composition through an appropriate diet, probiotic supplementation, and healthy lifestyle modifications. Such measures are important for maintaining the health of both the mother and the fetus during pregnancy [88]. In addition, studies suggest that glucose control achieved by insulin therapy in mothers with GDM may improve maternal gut microbiota composition, which could potentially influence the microbial profile transferred to the newborn [89]. Research conducted by Crussell et al. [83] showed that in infants born to mothers with GDM, the gut microbiota “catches up” by nine months of age to resemble that of infants born to healthy mothers. However, further studies are required to better understand how the microbiota of those infants evolves over time.

Further research on large prospective cohort studies involving mother–newborn pairs is needed for a more comprehensive understanding of the relationship between the microbiota and its direct influence on susceptibility to DM1 and DM2. Such research could provide critical insights into the development of effective preventive strategies in the future.

## 9. Discussion

One of the most common findings in both DM1 and DM2 is the reduced abundance of SCFA–producing bacteria (*Faecalibacterium prausnitzii*, *Roseburia)*. Bacterial metabolites, particularly SCFAs, play a crucial role in modulating metabolic pathways, including glucose and lipid metabolism. The decreased availability of SCFAs also exacerbates inflammation and intestinal barrier dysfunction. In both DM1 and DM2, dysbiosis can increase intestinal permeability, allowing proinflammatory bacterial LPS to enter the bloodstream. LPS is linked to autoimmunity in DM1 and insulin resistance in DM2. Another common finding in DM1, DM2, and GDM is the reduced abundance of bacteria belonging to the genus *Bifidobacterium*, which are generally considered beneficial members of the gut microbiota. A common feature of DM1, DM2, MODY, and GDM is the presence of gut microbiota dysbiosis.

A microbiota pattern that appears to be more characteristic of DM1 is the increased abundance of bacteria belonging to the phylum Bacteroidetes and the genus *Prevotella*, which may be associated with immune activation and the potential initiation of autoimmune processes. In contrast, microbiota alterations observed in DM2 are more closely linked to metabolic inflammation, insulin resistance and dyslipidemia. In particular, a decreased abundance of *Akkermansia* has been reported in patients with DM2 and appears to be associated with insulin resistance and impaired glucose metabolism. The most type-specific microbiota shift in GDM seems to be the reduced abundance of *Lactobacillus.* Changes characteristic of MODY are more difficult to identify due to the limited number of studies included in the analysis in our review.

It is important to note that some microbiota alterations attributed to diabetes may in fact be primarily driven by underlying metabolic disturbances rather than by the specific type of diabetes itself. The direction of this relationship remains unclear, as it is still debated whether dysbiosis contributes to the development of obesity and, consequently, diabetes, or whether obesity itself promotes the development of dysbiosis. In this review, we cited studies indicating that lower bacterial diversity is associated with insulin resistance and increased obesity. At the same time, experimental studies have demonstrated that intestinal LPS concentrations increase when mice are fed a high-fat diet. Some microbiome shifts that may be more strongly linked to obesity and dyslipidemia than to a specific diabetes type include those described mainly in the context of DM2. These alterations include reduced microbial diversity, decreased abundance of SCFA-producing bacteria, reduced levels of bacteria with BSH activity- such as *Lactobacillus*, *Bifidobacterium*, *Clostridium*, and *Bacteroides*- as well as decreased abundance of *Akkermansia* and an increased Firmicutes/Bacteroidetes ratio.

While studies indicate that gut dysbiosis is present in individuals already diagnosed with DM1, it remains unclear whether dysbiosis is a cause or a consequence of the disease. To gain a better understanding of the timing and nature of microbial changes preceding the clinical onset of DM1, tracking the microbiome composition from early infancy in genetically at-risk individuals is crucial.

Several limitations should be acknowledged that may significantly influence the data analyzed in this review. A large proportion of the available studies are cross-sectional and involve small sample sizes, which limits the ability to establish causal relationships. Differences in microbiome analysis approaches may lead to misleading or inconsistent outcomes due to the use of various sequencing methods and bioinformatics pipelines. More interventional studies, such as probiotic supplementation or dietary interventions, are needed to better clarify causal relationships, as associative data alone provide only speculative causal inferences.

This review also does not address potential interactions between medications, lifestyle factors, and geographic variability that may influence the observed outcomes. In particular, metformin has been shown to modulate gut microbiota composition, which may confound the interpretation of microbiota–disease associations [90]. Moreover, the cited studies are based on cohorts from diverse geographic regions spanning three continents: Europe, Asia, and North America. Therefore, differences in environmental exposures, socioeconomic factors, dietary habits, and genetic susceptibility should be considered as additional confounding factors. Importantly, diet represents a key determinant of gut microbiome composition and may influence the progression of diabetes, with its effects evident from early-life nutritional exposures. Breastfeeding vs. formula feeding, sugar intake and fibre intake represent only a few examples of variables that should be taken into consideration [91,92,93].

## 10. Future Directions

Future research should focus on establishing reproducible microbiota patterns that may serve as biomarkers for the risk of developing diabetes and disease progression, with priority given to longitudinal cohort studies. The most consistently reported patterns across different types of diabetes include a reduction in SCFA–producing bacteria, such as *Faecalibacterium prausnitzii* and *Roseburia* spp., as well as a decreased abundance of *Akkermansia muciniphila* and *Bifidobacterium* spp. However, inconsistencies between studies highlight the need for standardized microbiome profiling methodologies.

The most accessible, yet uncertain, preventive and therapeutic approach at present appears to be dietary intervention. While prebiotics, probiotics, and FMT are being studied and have shown initial beneficial effects, none of them are currently considered standard of care. High individual variability in microbiota composition, combined with the influence of environmental factors, limits its current clinical applicability. However, commonly used in diabetes, metformin has been shown to alter gut microbiota composition, suggesting that part of its therapeutic effect may be microbiota-related.

While the research is promising and opens up new possibilities for diabetes care and prevention, establishing a definitive causal relationship between dysbiosis and diabetes remains crucial, as does determining whether therapeutic strategies can be universal, reproducible, and applicable to large patient populations.

## 11. Conclusions

In recent years, numerous studies have examined the relationship between gut microbiota and various pathologies, including diabetes. The gut microbiota appears to have a significant influence on the pathophysiological mechanisms of diabetes. While several microbiota alterations appear to be shared across different types of diabetes, certain microbial patterns seem to be more type-specific. Nevertheless, confirming causal relationships, more longitudinal and interventional studies using standardized microbiome profiling methods are needed. Finally, establishing whether dysbiosis is a cause of diabetes or whether diabetes and its concomitant metabolic disturbances lead to dysbiosis remains a key research challenge. Table 2 summarizes the key studies cited in this review.

## Figures and Tables

**Table 1:** Gut microbiota differences and their functional impacts in DM1 and DM2.

Bacteria	DM1	DM2	Functional Impact
*Faecalibacterium prausnitzii*	↓	↓	Impaired glucose metabolism Insulin resistance Proinflammatory effects
*Roseburia intestinalis*	↓	↓	Impaired glucose metabolism
*Roseburia inulinivorans*	-	↓	-
Verrucomicrobiae class	-	↓	Insulin resistance
*Akkermansia* spp.	↓	↓ (*A. muciniphila* decrease may precede DM2 onset)	Insulin resistance Impaired glucose metabolism
*Bifidobacterium* spp.	↓↑ (mixed findings)	↓	Potential probiotic application
*Bacteroides* spp.	↑ (*B. ovatus*, *B. stercoris*, *B. fragilis*, *B. intestinalis*)	↑↓ (mixed findings, mostly increased)	Potential autoimmunity trigger in DM1 Proinflammatory effects
*Prevotella* spp.	↑	-	Proinflammatory effects Reduced gut barrier function
Firmicutes phylum	↓↑ (mixed findings)	↓ (increased Firmicutes/Bacteroidetes ratio)	Impaired glucose metabolism
Proteobacteria phylum	-	↑	Insulin resistance
*Lactobacillus* spp.	↓	↓↑ (mixed findings)	Impaired lipid metabolism
*Clostridium* spp.	-	↓	Impaired glucose/lipid metabolism
*Blautia* spp.	↑	↑	-
*Collinsella* spp.	↓	-	Abundance positively correlates with plasma insulin levels
Lachnospiraceae family	↑	-	Impaired glucose metabolism

Abbreviations: DM1, type 1 diabetes mellitus; DM2, type 2 diabetes mellitus; ↑, increased abundance; ↓, decreased abundance; -, data not reported or not applicable.

**Table 2:** Table summarizing key studies.

Study	Authors	Population/Model	Sample Size (N)	Main Findings
Functional and metabolic alterations of gut microbiota in children with new-onset type 1 diabetes [5]	Yuan X, Wang R, Han B, Sun C, Chen R, Wei H, et al.	children from China, mice	discovery cohort: 158; validation cohort: 65	DM1 is characterized by a distinct intestinal microbial profile, marked by increased LPS biosynthesis and decreased butyrate production and bile acid metabolism.
The human gut microbiome in early-onset type 1 diabetes from the TEDDY study [18]	Vatanen T, Franzosa EA, Schwager R, Tripathi S, Arthur TD, Vehik K, et al.	children from the United States and Finland, Germany, and Sweden	783	Microbiota alterations precede clinical disease onset. In DM1, early species-specific shifts act as potential triggers for autoimmunity.
Human Gut Microbiota Changes Reveal the Progression of Glucose Intolerance [36]	Zhang X, Shen D, Fang Z, Jie Z, Qiu X, Zhang C, et al.	men and women aged 46–64 years old from Beijing, China	121	Dysbiosis in gut microbiota occurs early before the prediabetes stage, which suggests that the proportion and diversity of microbiota can serve as potential makers for a high risk of diabetes.
The Gut Microbiota in Prediabetes and Diabetes: A Population-Based Cross-Sectional Study [37]	Wu H, Tremaroli V, Schmidt C, Lundqvist A, Olsson LM, Krämer M, et al.	men and women aged 50–64 years from the Gothenburg area, Sweden	discovery cohort: 1011; validation cohort: 484	It was observed that subjects with poor glucose tolerance had a lower amount of butyrate-producing bacteria in the composition of their gut microbiota, which is essential for regulating glucose metabolism.
Dietary intervention impact on gut microbial gene richness [48]	Cotillard A, Kennedy SP, Kong LC, Prifti E, Pons N, Le Chatelier E, et al.	overweight and obese individuals from France	49	Reduced gut microbiota diversity is associated with more pronounced metabolic disorders, including elevated levels of total cholesterol and triglycerides, insulin resistance, and increased inflammatory markers.
Increased Risk of Diabetes in Inflammatory Bowel Disease Patients: A Nationwide Population-based Study in Korea [75] Inflammatory Bowel Diseases Increase Risk of Type 2 Diabetes in a Nationwide Cohort Study [76]	Kang EA, Han K, Chun J, Soh H, Park S, Im JP, et al. Jess T, Jensen BW, Andersson M, Villumsen M, Allin KH	men and women aged 32–58 years with inflammatory bowel disease (IBD) and non-IBD controls from South Korea Danish population older than 30 years of age alive and residing in Denmark during 1977–2014	IBD group: 8070; control group: 40,350 cohort study: 6,028,844	Patients with Inflammatory Bowel Diseases have an increased risk of developing diabetes.
Comparative Studies of the Gut Microbiota in the Offspring of Mothers With and Without Gestational Diabetes [83]	Crusell MKW, Hansen TH, Nielsen T, Allin KH, Rühlemann MC, Damm P, et al.	A Danish cohort of 125 offspring born to mothers with and without GDM.	GDM group: 43; control group: 82	Children of mothers with GDM exhibited a differential composition of the gut microbiota both during the first week of life and at 9 months of age compared to the control group.

## Data Availability

No new data were created or analyzed in this study. Data sharing is not applicable to this article.

## Abbreviations

The following abbreviations are used in this manuscript:

DM1	Type 1 diabetes
DM2	Type 2 diabetes
MODY	Maturity-onset diabetes of the young
GDM	Gestational diabetes
HbA1c	Glycated hemoglobin
HDL	High-density lipoprotein cholesterol
SCFAs	Short-chain fatty acids
AMPK	AMP-activated protein kinase
GLUT4	Glucose transporter type 4
BCAAs	Branched-chain amino acids
FMT	Fecal microbiota transplantation
VLDL	Very-low-density lipoprotein
LDL	Low-density lipoprotein
TG	Triglycerides
GPR43	Free fatty acid receptor 2, FFA2
GPR41	Free fatty acid receptor 3, FFA 3
PYY	YY protein
FFAs	Free fatty acids
BSH	Bile salt hydrolase
FXR	Farnesoid X receptor
TGR5	Takeda G protein-coupled receptor 5
GLP-1	Glucagon-like peptide-1
LGS	Leaky gut syndrome
S-IgA	Secretory immunoglobulin A
NOD2	Nucleotide-binding oligomerization domain 2
XBP1	X-box binding protein 1
TJ	Tight junction
Treg	T regulatory lymphocytes
LPS	Lipopolysaccharide
TLR	Toll-like receptor
IL-1	Interleukin 1
IL-6	Interleukin 6
TNF-α	Tumor necrosis factor alpha
IRS-1	Insulin receptor substrate-1
hsCRP	High-sensitivity C-reactive protein
SDS	Sodium Dodecyl Sulfate
